# Impact of hyperhydration on the mortality risk in critically ill patients admitted in intensive care units: comparison between bioelectrical impedance vector analysis and cumulative fluid balance recording

**DOI:** 10.1186/s13054-016-1269-6

**Published:** 2016-04-08

**Authors:** Sara Samoni, Valentina Vigo, Luis Ignacio Bonilla Reséndiz, Gianluca Villa, Silvia De Rosa, Federico Nalesso, Fiorenza Ferrari, Mario Meola, Alessandra Brendolan, Paolo Malacarne, Francesco Forfori, Raffaele Bonato, Carlo Donadio, Claudio Ronco

**Affiliations:** Institute of Life Sciences, Sant’Anna School of Advanced Studies, Pisa, Italy; Department of Nephrology, Dialysis and Transplantation, San Bortolo Hospital, International Renal Research Institute Vicenza (IRRIV), Viale Rodolfi, 37, 36100 Vicenza, Italy; Department of Clinical & Experimental Medicine, University of Pisa, Pisa, Italy; Department of Anaesthesiology and Intensive Care, San Bortolo Hospital, Vicenza, Italy; Department of Anaesthesia and Intensive Care Unit 6, Cisanello Hospital, Pisa, Italy; Department of Anaesthesia and Intensive Care Unit 4, Cisanello Hospital, Pisa, Italy

**Keywords:** Bioelectrical impedance vector analysis, Cumulative fluid balance, Fluid overload, Hyperhydration, Intensive care unit, Mortality

## Abstract

**Background:**

Studies have demonstrated a positive correlation between fluid overload (FO) and adverse outcomes in critically ill patients. The present study aims at defining the impact of hyperhydration on the Intensive Care Unit (ICU) mortality risk, comparing Bioelectrical Impedance Vector Analysis (BIVA) assessment with cumulative fluid balance (CFB) recording.

**Methods:**

We performed a prospective, dual-centre, clinician-blinded, observational study of consecutive patients admitted to ICU with an expected length of ICU stay of at least 72 hours. During observational period (72–120 hours), CFB was recorded and cumulative FO was calculated. At the admission and daily during the observational period, BIVA was performed. We considered FO between 5 % and 9.99 % as moderate and a FO ≥10 % as severe. According to BIVA hydration scale of lean body mass, patients were classified as normohydrated (>72.7 %–74.3 %), mild (>71 %–72.7 %), moderate (>69 %–71 %) and severe (≤69 %) dehydrated and mild (>74.3 %–81 %), moderate (>81 %–87 %) and severe (>87 %) hyperhydrated. Two multivariate logistic regression models were performed: the ICU mortality was the response variable, while the predictor variables were hyperhydration, measured by BIVA (BIVA model), and FO (FO model). A *p*-value <0.05 was considered to indicate statistical significance.

**Results:**

One hundred and twenty-five patients were enrolled (mean age 64.8 ± 16.0 years, 65.6 % male). Five hundred and fifteen BIVA measurements were performed. The mean CFB recorded at the end of the observational period was 2.7 ± 4.1 L, while the maximum hydration of lean body mass estimated by BIVA was 83.67 ± 6.39 %. Severe hyperhydration measured by BIVA was the only variable found to be significantly associated with ICU mortality (OR 22.91; 95 % CI 2.38–220.07; *p* < 0.01).

**Conclusions:**

The hydration status measured by BIVA seems to predict mortality risk in ICU patients better than the conventional method of fluid balance recording. Moreover, it appears to be safe, easy to use and adequate for bedside evaluation. Randomized clinical trials with an adequate sample size are needed to validate the diagnostic properties of BIVA in the goal-directed fluid management of critically ill patients in ICU.

## Background

Accurate fluid management in patients admitted to an intensive care unit (ICU) is one of the most challenging and important tasks for both intensivists and nephrologists. Despite progress in the standard intensive care, the assessment of hydration status and consequent treatment are still complex and require an in-depth knowledge of body fluid homeostasis. Several studies have demonstrated a positive correlation between fluid overload (FO) and adverse outcomes in critically ill patients [1–12]. In particular, FO increases the length of mechanical ventilation [1], ICU stay [1] and incidence of acute kidney injury (AKI) [2] in patients with acute lung injury; it increases incidence of AKI [3] and need of renal replacement therapy (RRT) [3] in septic patients; it worsens recovery of renal function [4, 5] in patients with AKI; it decreases RRT-free days [6] in continuous RRT (CRRT) patients; and it increases the incidence of infectious complications in surgical patients [7] and of intra-abdominal hypertension in patients admitted to ICU for all causes [8]. In addition, FO is associated with an increase in mortality in all the above-mentioned patient categories [3, 4, 6–12].

Current studies define patients’ hydration status on the basis of body weight variations during ICU stay, registered fluid balance or both. It is noteworthy that precise body weight may be difficult to measure in ICU patients and its value may be affected by body composition changes due to other reasons than fluid administration [13]. On the other hand, the registration of fluid balance by the difference of inputs and outputs of fluids does not usually consider insensible losses and has shown low accuracy [14, 15], suggesting the need of more accurate tools.

Bioelectrical impedance vector analysis (BIVA) measures total body impedance which derives from resistance (R) and reactance (Xc). R and Xc represent the opposition to an alternating electric flow exerted, respectively, by the intra- and extra-cellular electrolyte solutions and by the interfaces of cell membranes and tissues. The two measurements, standardized for height, identify the status of hydration and soft tissue mass. This technique has been validated in healthy individuals [16, 17] as well as in maintenance haemodialysis and peritoneal dialysis patients [18–21]. In spite of previous studies reporting unclear and controversial evidence about the effectiveness of bioimpedance in critically ill patients [22–24], the modern BIVA technique seems to be reliable, especially to detect hydration changes in repeated measurements.

The aim of the present study is to assess the impact of hyperhydration on ICU mortality in critically ill patients, comparing its measurement by BIVA and by cumulative fluid balance (CFB) recording.

## Methods

### Design, setting and patients

We performed a prospective dual-centre, clinician-blinded, observational study with the approval of the Institutional Review Boards of San Bortolo Hospital in Vicenza and of the University Hospital in Pisa, Italy. The requirement to obtain informed consent was waived because bioimpedance was performed in both ICUs participating in the study, as routine evaluation of critically ill patients. This study was performed according to the ethical principles of the Declaration of Helsinki. Inclusion criteria were adult patients newly admitted to the ICU of participating centres older than 18 years and with an expected length of stay in ICU of 72 hours or more, as judged by clinicians. Exclusion criteria were: (1) pregnancy, (2) limb amputations, (3) multi-drug resistance infection and (4) therapy with an extra-corporeal membrane oxygenator. The observational period started within 24 hours from admission to ICU and continued for at least 72 hours to a maximum of 120 hours.

### Data collection and management

Demographics, anthropometrics, comorbidities information and causes of hospitalization were recorded for each patient into study-specific case report forms and a database. Acute physiology and chronic health evaluation (APACHE) II and simplified acute physiology score (SAPS) II were determined during the first 24 hours after ICU admission; sequential organ failure assessment (SOFA) was calculated daily. Additional variables including clinical data (e.g. arterial blood pressure, heart and respiratory rate and body temperature), laboratory data and details of hospital course (e.g. use and dosage of vasopressor agents, need for mechanical ventilation and for CRRT) were recorded at admission and every day during the observation period. The occurrences of AKI and septic shock were diagnosed on the basis of Kidney Disease: Improving Global Outcomes (KDIGO) criteria [25] and surviving sepsis campaign (SSC) criteria [26], respectively.

Daily fluid balance was recorded as the algebraic sum of fluid intake and output per day, not including insensible losses, while CFB was calculated as the algebraic sum of daily fluid balance during the observational period. Cumulative fluid overload was calculated by dividing the CFB by the admission weight of each patient; the result was expressed as a percentage. We considered a FO between 5 % and 9.99 % as moderate and a FO ≥10 % as severe. While the definition of FO as a percentage of fluid accumulation ≥10 % over baseline body weight at hospital admission is based upon published literature [4], we arbitrarily proposed a further classification into moderate and severe FO, as described above.

The assessments of hydration status were performed using a single frequency electrical impedance analyser (RenalEFG, Akern, Firenze, Italy) during the first 24 hours after ICU admission and daily for a period of 72–120 hours. BIVA parameters collected were: (1) R, (2) Xc, (3) phase angle and (4) hydration percentage of lean body mass. They were measured by an alternating electric flow of 300 microA and an operating frequency of 50 kHz. BIVA analyses were performed by three different trained operators, with the patient in the supine position on the hospital bed without touching metal objects. The angles between upper limbs and trunk and between the legs were 30 and 45°, respectively, according to the manufacturer’s indications. The skin was cleaned with alcohol or saline before the application of electrodes on the right hand and foot. Patients’ hydration status was classified into three main categories: normohydrated, dehydrated and hyperhydrated. According to the numerical scale for BIVA, the normal level of hydration was set between >72.7 % and 74.3 % of lean body mass (class 0). Higher and lower values represented states of hyperhydration and dehydration, respectively. Dehydration was classified into mild (class -1: >71 %–72.7 %), moderate (class -2: >69 %–71 %) and severe (class -3: ≤69 %). Similarly, hyperhydration was classified into mild (class +1: >74.3 %–81 %), moderate (class +2: >81 %–87 %) and severe (class +3: >87 %) [27]. BIVA results were not made available to treating clinicians at any time during the study.

### Statistical analysis

Data are expressed as means ± standard deviations, medians and interquartile ranges or frequency distributions, as appropriate. The trend of hydration status during the observational period was described through box-plots. The relationship between FO and the different classes of hydration, obtained using BIVA, was described through a box-plot. The impact of hyperhydration estimated by BIVA on the overall survival, defined as number of days of life from the date of ICU admission until death or the end of the study, was described by a Kaplan-Meier survival curve. At the end of the study, all patients were discharged from ICU.

The relationship between ICU mortality and classes of hydration was analysed through a multivariate logistic regression model where the response variable was ICU mortality and the predictor variables were the presence of moderate and severe hyperhydration and all other available variables (BIVA model). The BIVA measurements were aggregated by taking the maximum value observed for each patient. The relationship between ICU mortality and moderate and severe FO calculated on the basis of CFB was analysed through a multivariate logistic regression model where the response variable was ICU mortality and the predictor variables were FO between 5 % and 9.99 % and FO equal to or greater than 10 % and all other available variables (FO models). Since we found that mild hyperhydration was not correlated with a higher mortality risk, the initial BIVA model was performed with ICU mortality as the response variable and the presence of moderate and severe hyperhydration measured by BIVA and all other characteristics available as predictor variables. Then, the initial model was modified according to a step-wise analysis that progressively deleted non-significant variables (*p* value >0.1). The same predictor variables were used to perform the FO model with FO between 5 % and 9.99 % and FO equal to or greater than 10 % instead of classes of hyperhydration. A *p*-value <0.05 was considered to indicate statistical significance.

Statistical analysis was performed using “R” software (R Core Team (2015). R: a language and environment for statistical computing. Foundation for Statistical Computing, Vienna, Austria. URL https://www.r-project.org/).

## Results

### Description of study population

A total of 125 patients were enrolled in the study from 25 May 2012 to 15 January 2015. Since patients undergoing multiple invasive procedures could not be included, as their observation period could not start within the first 24 hours, and since we did not include patients admitted on weekends or during holidays, as BIVA measurements were performed only by three operators in two centres, we achieved a low recruitment rate. The main characteristics of the study population at admission and during ICU stay are shown in Table 1. The mean age of the patients enrolled was 64.8 ± 16.0 years, 65.6 % of the patients were male. The most frequent criteria for ICU admission were post-surgical monitoring (31.2 %), trauma (28.8 %), stroke (28 %) and sepsis (26.4 %) (Table 1). AKI, defined according to KDIGO criteria [25], and septic shock, as described by SSC criteria [26], occurred respectively in 30.4 % and 16 % of patients, while CRRT was performed in 16.8 % of cases (Table 1). The overall survival was 63.2 %. During ICU stay, 28 patients (22.4 %) died, 13 patients (10.4 %) died after ICU discharge during hospital stay and 5 patients (4 %) died after hospital discharge.

**Table 1:** Main characteristics of study population at admission in intensive care unit

Demographic, anthropometric data and comorbidities	Entire cohort (*n* = 125)
Sex (male)	82 (65.6)
Age (years)	64.78 ± 15.96; [68 (21)]
Height (m)	1.71 ± 0.10; [1.72 (0.13)]
CKD 1–4	19 (15.2)
CKD 5	3 (2.4)
Dialysis	3 (2.4)
Diabetes	25 (20)
Arterial hypertension	71 (56.8)
Coronary artery disease	22 (17.6)
COPD	12 (9.6)
Cirrhosis	4 (3.2)
Cancer	20 (16)
Admission diagnosis	Entire cohort (*n* = 125)
Sepsis	33 (26.4)
Post-surgery	39 (31.2)
Trauma	36 (28.8)
Cardiac arrest	9 (7.2)
Heart failure	24 (19.2)
Stroke	35 (28)
Clinical data at ICU admission	Entire Cohort (*n* = 125)
Mechanical ventilation	111 (88.8)
FiO_2_	0.47 ± 0.14; [0.40 (0.10)]
Arterial pH	7.44 ± 0.11; [7.45 (0.10)]
Serum potassium (mEq/L)	3.86 ± 0.62; [3.87 (0.8)]
Serum bicarbonate (mmol/L)	25.36 ± 5.23; [25.4 (5.3)]
Serum creatinine (mg/dL)	1.38 ± 1.32; [0.93 (0.87)]
Serum urea (mg/dL)	60.53 ± 53.15; [45 (39)]
Systolic BP (mmHg)	129.83 ± 33.56; [124 (54)]
Diastolic BP (mmHg)	59.5 ± 15.8; [58 (21)]
Mean arterial pressure (mmHg)	82.94 ± 19.42; [80 (26.67)]
CVP (mmHg)	10.18 ± 3.52; [10 (10)]
Heart rate	80.88 ± 23.2; [79 (32)]
Haemoglobin (g/dL)	10.67 ± 2.14; [10.6 (3.12)]
White blood cells (10^3^/mL)	13315.67 ± 14513.79; [10930 (5400)]
ICU scoring system	Entire Cohort (*n* = 125)
APACHE II score	19.28 ± 6.88; [17 (10)]
SAPS II score	50.03 ± 16.8; [50 (22)]
SOFA score at ICU admission	9.24 ± 4.2; [8 (5)]
Clinical data during ICU stay	Entire cohort (*n* = 125)
AKI	38 (30.4)
CRRT	21 (16.8)
Septic shock	20 (16)

Data are expressed as means (SD); [medians (interquartile range)] or number (percent). *CKD* chronic kidney disease, *COPD* chronic obstructive pulmonary disease, *ICU* intensive care unit, *FiO*_*2*_ fraction of inspired oxygen, *BP* blood pressure, *CVP* central venous pressure, *APACHE II* acute physiology and chronic health evaluation II, *SAPS II* simplified acute physiology score II, *SOFA* sequential organ failure assessment, *AKI* acute kidney injury, *CRRT* continuous renal replacement therapy

**Fig. 1:**
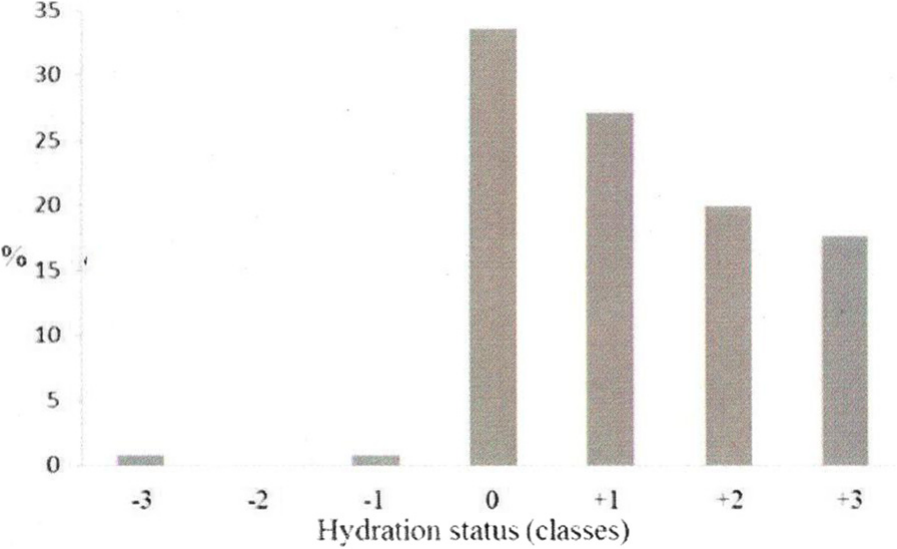
Distribution of hydration status, in classes, at admission in intensive care unit. Classes of hydration status are defined, according to a numerical scale for BIVA as follow: -3) severe dehydration (≤69 %), -2) moderate dehydration (>69 %–71 %), -1) mild dehydration (>71 %–72.7 %), 0) normohydration (>72.7 %–74.3 %), +1) mild hyperhydration (>74.3 %–81 %), +2) moderate hyperhydration (>81 %–87 %) +3) severe hyperhydration (>87 %). *BIVA* bioelectric impedance vector analysis

**Fig. 2:**
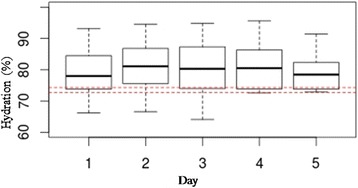
Box-plot of daily BIVA hydration values during the observation period. The *horizontal lines* represent the minimum value, first quartile, median, third quartile and maximum value. The *dashed lines* correspond to accepted limits of normohydration. *BIVA* bioelectric impedance vector analysis

**Fig. 3:**
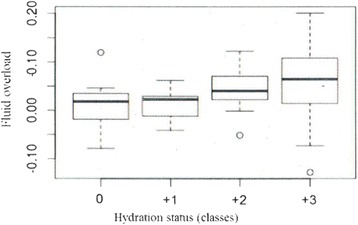
Box-plot of trend of fluid overload in the different classes of hydration. Classes of hydration status are defined, according to a numerical scale for BIVA as follow: 0) normohydration (>72.7 %–74.3 %), +1) mild hyperhydration (>74.3 %–81 %), +2) moderate hyperhydration (>81 %–87 %), +3) severe hyperhydration (>87 %). The *horizontal lines* represent the minimum value, first quartile, median, third quartile and maximum value. *BIVA* bioelectric impedance vector analysis

A total of 515 BIVA measurements were performed. According to the previously mentioned categories, 64.8 % of patients were hyperhydrated, 33.6 % normohydrated and only 1.6 % dehydrated at ICU admission. In particular, 0.8 % of patients were mildly, 0 % moderately and 0.8 % severely dehydrated, while 27.2 % were mildly, 20 % were moderately and 17.6 % were severely hyperhydrated (Fig. 1). Patients’ hydration status remained above normal values for all observation periods (Fig. 2). In fact, while at enrolment there were 42 patients with normal and 83 with abnormal BIVA values, including 81 hyperhydrated and 2 dehydrated individuals, by the end of the observation period, the number of patients enrolled with normal BIVA values who remained normal was 20, whereas of those with abnormal BIVA values at enrolment, 17 had reverted to normal. Concerning fluid status at the end of the observation period, the mean CFB recorded was 2.7 ± 4.1 L, while the mean hydration of lean body mass estimated by BIVA was 80.68 ± 5.82 %. Patients in higher classes of hydration had a greater FO than patients in lower classes and they were more likely to have FO of more than 5 %. Indeed, in normohydrated patients, the median CFB was 1.8 % of body weight, while in severely hyperhydrated patients it was 6.4 % (Fig. 3).

### Outcomes

**Fig. 4:**
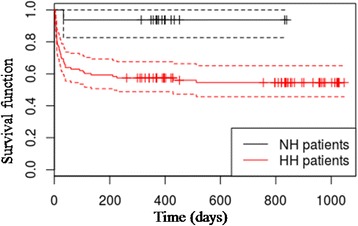
Kaplan-Meier survival curves showing relation between hydration status and long-term mortality. The vertical lines represent censored subjects. The follow-up duration is different for each subject because it is censored at the end of the study. Patients were defined as hyperhydrated (HH) if they overreached the value of 74.3 % of lean body mass at least once during the observation period. *NH* normohydrated, *HH* hyperhydrated, time (days)

Taking into account the impact of hyperhydration estimated by BIVA on the overall survival, defined as number of days of life from the date of ICU admission until death or the end of the study, the Kaplan-Meier survival curves show a greater mortality in hyperhydrated than in normohydrated patients (Fig. 4).

**Fig. 5:**
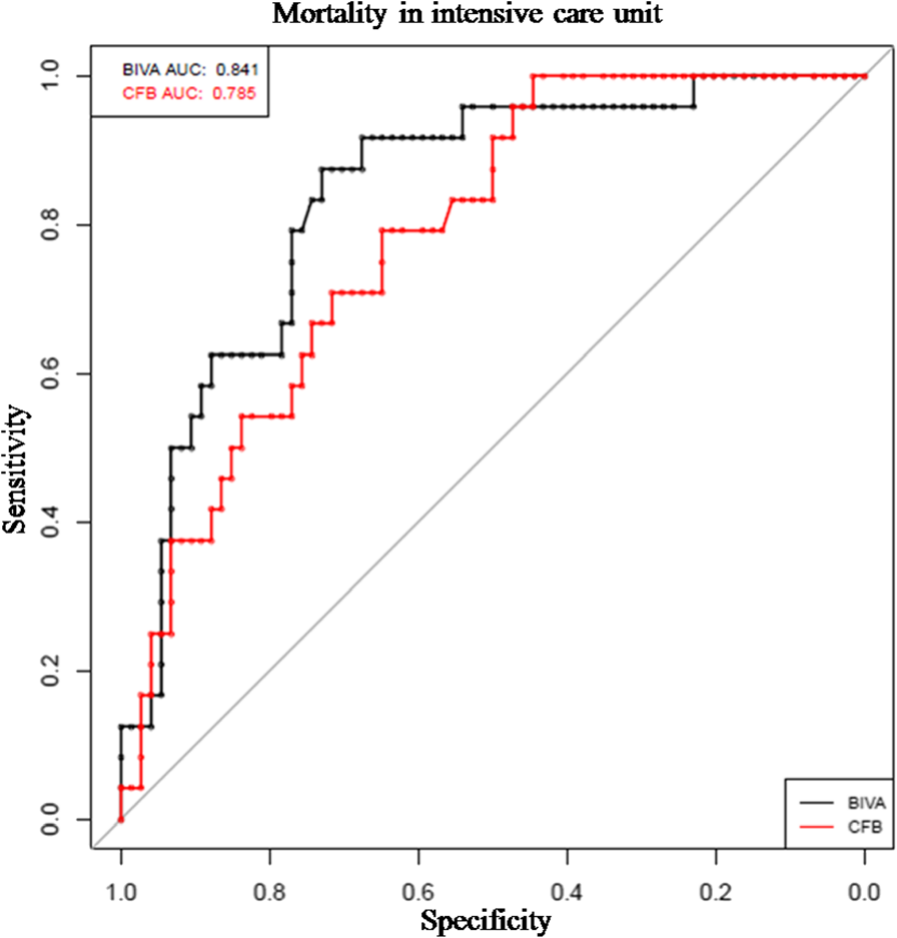
ROC curves for the two models. *BIVA* bioelectrical impedance vector analysis, *CFB* cumulative fluid balance; *AUC* area under the ROC curve, *ROC* receiver operating characteristic

According to the final BIVA model, shown in Table 2, we found a significant association between ICU mortality and severe hyperhydration measured by BIVA (OR 22.91; 95 % CI 2.38–220.07; *p* < 0.01). The final logistic model correctly classifies 21 of 24 deaths occurred in ICU (sensitivity = 87.5 %) and 52 of 74 survivors at ICU discharge (specificity = 70.3 %), with the optimal threshold of 24 % (i.e. classifying as dead those for whom, through the logistic model, we estimate a probability of death greater than or equal to 24 %). The FO model failed to show any significant association between FO and ICU mortality. Moreover, the sensitivity and the specificity of the FO model on estimating probability of death were less than the previous one: sensitivity 66.7 % and specificity 66.2 %, taking into account a FO equal to or greater than 10 %. Furthermore, the BIVA model showed an Akaike Information Criterion (AIC) lower than the FO model and the areas under the ROC curves (AUC) were, respectively, 0.841 and 0.785 for BIVA and the FO model (Fig. 5).

**Table 2:** BIVA model

	Coef	OR	95 % CI		*p* value
			lower	upper	
Stroke as ICU admission diagnosis	1.636	5.138	1.293	20.417	0.020*
Hypertension	1.084	2.956	0.894	9.774	0.075
COPD	-2.333	0.096	0.012	0.766	0.026*
CRRT in ICU stay	1.257	3.515	0.937	13.185	0.062
SAPS II	0.040	1.041	1.003	1.080	0.032*
Maximum hydration of lean body mass 81–87 %	2.005	7.426	0.746	73.913	0.087
Maximum hydration of lean body mass >87 %	3.131	22.913	2.385	220.077	0.006**

Logistic analysis with mortality in the intensive care unit as response variable and the presence of moderate and severe hyperhydration as predictor variables. *p ≤ 0.05; **p ≤ 0.01; ***p ≤ 0.001. AIC: 95.372. *Coef* coefficient, *OR* odds ratio, *CI* confidence interval, *ICU* intensive care unit, *COPD* chronic obstructive pulmonary disease*, CRRT* continuous renal replacement therapy, *SAPS II* simplified acute physiology score II, *AIC* Akaike information criterion

## Discussion

The impact of FO on mortality in critically ill patients has been described in several clinical studies [3, 4, 6–12]. In all of them, hydration status was evaluated by means of fluid balance recording or body weight measurement. Our results are consistent with these findings: providing an objective measure of FO. Indeed, in contrast to previous studies that reported controversial evidence about the effectiveness of bioimpedance in ICU patients [22–24], we found a significant correlation between hyperhydration measured by BIVA and long-term mortality in patients admitted to ICU for all causes, with or without AKI (Fig. 4).

To compare BIVA and CFB recording in the assessment of hydration status and, thus, in predicting mortality in ICU patients, we performed two multivariate logistic regression models with ICU mortality as response variable: in the BIVA model, we evaluated the association between ICU mortality and the presence of moderate and severe hyperhydration; in the FO model the predictor variables tested were FO between 5 % and 9.99 % and FO ≥10 %. Severe hyperhydration measured by BIVA was the only variable found to be significantly associated with ICU mortality (OR 22.91; 95 % CI 2.38–220.07; *p* < 0.01). Moreover, all indexes (AIC, sensitivity and specificity) indicate the superiority of the BIVA model when compared with the other model (Tables 2, 3). Physical examination, daily assessment of fluid balance and/or body weight represents the common clinical practice regarding decision making on fluid administration and diuretic therapy in critical care patients. However, clinical signs may be delayed [28] and the accuracy and the reliability of fluid balance recording and body weight measurement in ICU patients has already been questioned [14, 15]. While the amount of administered fluid is well known, the output may be difficult to compute accurately; indeed, even when insensible losses are taken into account, they depend on several factors (i.e. time, body temperature, room temperature and humidity) and may also be difficult to measure.

**Table 3:** FO model

	Coef	OR	95 % CI		*p* value
			lower	upper	
Stroke as ICU admission diagnosis	1.241	3.459	0.986	12.126	0.052
Hypertension	0.940	2.560	0.839	7.808	0.098
COPD	-1.760	0.172	0.019	1.530	0.114
CRRT in ICU stay	1.426	4.162	1.159	14.938	0.028*
SAPS II	0.034	1.035	0.998	1.071	0.060
5 % < FO ≤ 9.99 %	0.465	1.592	0.447	5.658	0.472
FO ≥ 10 %	1.131	3.099	0.743	12.908	0.120

Logistic analysis with ICU mortality as response variable and FO between 5 % and 9.99 % and ≥ 10 % as predictor variables. *p ≤ 0.05; **p ≤ 0.01; ***p ≤ 0.001. AIC: 106.05. *Coef* coefficient, *OR* odds ratio, *CI* confidence interval, *ICU* intensive care unit, *COPD* chronic obstructive pulmonary disease, *CRRT* continuous renal replacement therapy, *SAPS II* simplified acute physiology score II, *FO* fluid overload

At present, the gold-standard method to evaluate fluid assessment is isotope dilution, but it is difficult to perform in critical care patients because of fluid sequestration and abnormal penetration of tracers into cells [29]. Even though BIVA is commonly used in healthy subjects [16, 17] and in patients suffering from kidney disease [18–21], both in haemodialysis and in peritoneal dialysis, its role in critically ill patients is still controversial [22–24]. Bioelectrical parameters (R, Xc), indexed to height, are graphically represented in a normogram: RXc graph simultaneously describes hydration status and soft tissue mass compared to the standard deviation ellipses. Taking into account the major axis of the graph, a shorter resulting vector identifies a higher content of body fluids, reaching extremes out of the pole [16]. In order to allow a simpler interpretation, an algorithm was developed to finally convert these parameters into a synthetic measure of lean body mass hydration percentage. According to this numerical scale, patients can be classified as dehydrated, normohydrated and hyperhydrated [27]. In a recent study, the relationship between BIVA hydration and changes in fluid balance was assessed in 61 critically ill patients. Similar to our study, results showed an increase in BIVA-measured hydration in patients with calculated fluid accumulations > 1 L. A statistically significant decrease in BIVA hydration was found in parallel with median fluid loss of 2.4 L. This study supported the correlation of BIVA with CFB over the time in ICU suggesting the use of BIVA as a guide for fluid management in critically ill patients [30].

Although BIVA can assess intracellular and extracellular fluid, it cannot discern between extravascular and intravascular volume. Despite that in healthy subjects there is equilibrium among body spaces, in ICU patients several clinical settings may lead to disorders in body fluid distribution balance (mechanical ventilation, malnourishment, sepsis, heart disease, and so on), thus potentially creating distrust of the use of BIVA in ICU patients. We must emphasise that bioimpedance does not predict fluid-responsiveness, but it assesses hydration status and, according to our results, mortality risk in patients admitted to ICU. In patients with acute inflammatory insults, proinflammatory cytokines and hormones determine arterial vasodilation and transcapillary albumin leak, leading to arterial underfilling, microcirculatory dysfunction and secondary interstitial oedema with systemic hypoperfusion. Later, compensatory neuroendocrine reflexes and potential renal dysfunction induce hydrosaline retention and FO [13]. While an early adequate fluid repletion is required in these patients to prevent a multiple organ dysfunction syndrome, authors have recently underlined the importance of a correct late fluid management [31, 8]. Nevertheless, despite all evidence about the correlation between FO and adverse outcomes, correct fluid management in critically ill patients is still far off. Indeed, in our cohort, hyperhydration was highly prevalent, probably due to fluids administered at hospital admission, and it persisted during the whole ICU stay in almost all patients.

There are several limitations to our study: 1) we studied a population including both surgical and medical ICU patients; the a priori decision was made to recruit a heterogeneous population to test our concept, but its performance will have to be validated externally and in niche populations; 2) our electronic data capture system does not include an automatic estimation of insensible losses; 3) we did not distinguish the types of administered fluids; and 4) our study only considered the hydration percentage of lean body mass; nevertheless, it has been well described that in critical illness proteolysis autophagy results in rapid loss of muscle mass that may affect the estimation of other parameters of body composition.

Regarding the high prevalence of hyperhydration in our cohort of patients, but also its correlation with mortality risk and the high feasibility of BIVA, we believe that the routine use of BIVA may help physicians to individuate patients’ ideal body weights and may drive fluid administration and/or diuretic therapy.

## Conclusions

Our findings confirm and expand literature data about the correlation between hyperhydration and ICU mortality. Despite the importance of this problem, there are currently few non-invasive methods to assess hydration status in critically ill patients. The hydration scale of lean body mass, obtained by means of BIVA, seems to predict mortality risk in ICU patients better than the conventional method of fluid balance recording. Moreover, impedance analysis has been shown to be safe, easy to use and adequate for bedside evaluation. Randomized clinical trials with an adequate sample size are needed to validate the diagnostic properties of BIVA in the goal-directed fluid management of critically ill patients in ICU.

## Key messages

- In our cohort, hyperhydration was highly prevalent and it persisted during the entire ICU stay in almost all patients.
- A significant correlation was found between hyperhydration measured by BIVA and long-term mortality in patients admitted to ICU for all causes, with or without AKI.
- Severe hyperhydration measured by BIVA was the only variable found to be significantly associated with ICU mortality, when compared to CFB recording.

## Abbreviations

AIC: Akaike information criterion
AKI: Acute kidney injury
APACHE II: Acute physiology and chronic health evaluation II
AUC: Area under the ROC curve
BIVA: Bioelectrical impedance vector analysis
BP: Blood pressure
CFB: Cumulative fluid balance
CKD: Chronic kidney disease
COPD: Chronic obstructive pulmonary disease
CRRT: Continuous RRT
CVP: Central venous pressure
FiO_2_: Fraction of inspired oxygen
FO: Fluid overload
ICU: Intensive care unit
R: Resistance
ROC: Receiver operating characteristic
RRT: Renal replacement therapy
SAPS II: Simplified acute physiology score II
SOFA: Sequential organ failure assessment
SSC: Surviving sepsis campaign
Xc: Reactance

## References

[1] Wiedemann HP, Wheeler AP, Bernard GR, Thompson BT, Hayden D, de Boisblanc B, Connors AF, Hite RD, Harabin AL. Comparison of two fluid-management strategies in acute lung injury. N Engl J Med. 2006;354:2564–75. 10.1056/NEJMoa062200 16714767

[2] Liu KD, Thompson BT, Ancukiewicz M, Steingrub JS, Douglas IS, Matthay MA, Wright P, Peterson MW, Rock P, Hyzy RC, Anzueto A, Truwit JD; National Institutes of Health National Heart, Lung and BIARDSN. Acute kidney injury in patients with acute lung injury: impact of fluid accumulation on classification of acute kidney injury and associated outcomes. Crit Care Med. 2012;39:2665–71. 10.1097/CCM.0b013e318228234b PMC3220741 21785346

[3] Payen D, de Pont AC, Sakr Y, Spies C, Reinhart K, Vincent JL (2008). A positive fluid balance is associated with a worse outcome in patients with acute renal failure. Crit Care.

[4] Bouchard J, Soroko SB, Chertow GM, Himmelfarb J, Ikizler TA, Paganini EP, Mehta RL. Fluid accumulation, survival and recovery of kidney function in critically ill patients with acute kidney injury. Kidney Int. 2009;76:422–7. 10.1038/ki.2009.159 19436332

[5] Heung M, Wolfgram DF, Kommareddi M, Hu Y, Song PX, Ojo AO (2012). Fluid overload at initiation of renal replacement therapy is associated with lack of renal recovery in patients with acute kidney injury. Nephrol Dial Transplant.

[6] Bellomo R, Cass A, Cole L, Finfer S, Gallagher M, Lee J, Lo S, McArthur C, McGuiness S, Norton R, Myburgh J, Scheinkestel C, Su S. An observational study fluid balance and patient outcomes in the Randomized Evaluation of Normal vs. Augmented Level of Replacement Therapy trial. Crit Care Med. 2012;40:1753–60. 10.1097/CCM.0b013e318246b9c6 22610181

[7] Barmparas G, Liou D, Lee D, Fierro N, Bloom M, Ley E, Salim A, Bukur M. Impact of positive fluid balance on critically ill surgical patients: a prospective observational study. J Crit Care. 2014;29:936–41. 10.1016/j.jcrc.2014.06.023 25085510

[8] Malbrain ML, Marik PE, Witters I, Cordemans C, Kirkpatrick AW, Roberts DJ, Regenmortel N Van. Fluid overload, de-resuscitation, and outcomes in critically ill or injured patients: a systematic review with suggestions for clinical practice. Anaesthesiol Intensive Ther. 2014;46:361–80. 10.5603/AIT.2014.0060 25432556

[9] Vincent JL, Sakr Y, Sprung CL, Ranieri VM, Reinhart K, Gerlach H, Moreno R, Carlet J, Le Gall J-R, Payen D. Sepsis in European intensive care units: results of the SOAP study. Crit Care Med. 2006;34:344–53. 10.1097/01.ccm.0000194725.48928.3a 16424713

[10] Vaara ST, Korhonen AM, Kaukonen KM, Nisula S, Inkinen O, Hoppu S, Laurila JJ, Mildh L, Reinikainen M, Lund V, Parviainen I, Pettila V, Finnaki SG. Fluid overload is associated with an increased risk for 90-day mortality in critically ill patients with renal replacement therapy: data from the prospective FINNAKI study. Crit Care. 2012;16:R197. 10.1186/cc11682 PMC3682299 23075459

[11] Boyd J, Forbes J, Nakada T, Walley K, Russell J (2011). Fluid resuscitation in septic shock: a positive fluid balance and elevated central venous pressure are associated with increased mortality. Crit Care Med.

[12] Wang N, Jiang L, Zhu B, Wen Y, Xi XM (2015). Fluid balance and mortality in critically ill patients with acute kidney injury: a multicenter prospective epidemiological study. Crit Care.

[13] Cordemans C, De Laet I, Van Regenmortel N, Schoonheydt K, Dits H, Huber W, Malbrain ML. Fluid management in critically ill patients: the role of extravascular lung water, abdominal hypertension, capillary leak, and fluid balance. Ann Intensive Care. 2012;2(Suppl 1 Diagnosis and management of intra-abdominal hyperten):S1. 10.1186/2110-5820-2-S1-S1 PMC3390304 22873410

[14] Perren A, Markmann M, Merlani G, Marone C, Merlani P (2011). Fluid balance in critically ill patients. Should we really rely on it?. Minerva Anestesiol.

[15] Eastwood GM (2006). Evaluating the reliability of recorded fluid balance to approximate body weight change in patients undergoing cardiac surgery. Heart Lung.

[16] Piccoli A, Rossi B, Pillon L, Bucciante G (1994). A new method for monitoring body fluid variation by bioimpedance analysis: the RXc graph. Kidney Int.

[17] Piccoli A, Nigrelli S, Caberlotto A, Bottazzo S, Rossi B, Pillon L, Maggiore Q. Bivariate normal values of the bioelectrical impedance vector in adult and elderly populations. Am J Clin Nutr. 1995;61:269–70. 10.1093/ajcn/61.2.269 7840061

[18] Piccoli A (1998). Identification of operational clues to dry weight prescription in hemodialysis using bioimpedance vector analysis. Kidney Int.

[19] Pillon L, Piccoli A, Lowrie EG, Lazarus JM (2004). Vector length as a proxy for the adequacy of ultrafiltration in hemodialysis using bioimpedance vector analysis. Kidney Int.

[20] Piccoli A (2004). Bioelectric impedance vector distribution in peritoneal dialysis patients with different hydration status. Kidney Int.

[21] Donadio C, Consani C, Ardini M, Bernabini G, Caprio F, Grassi G, Lucchesi A, Nerucci B. Estimate of body water compartments and body composition in maintenance hemodialysis patients. J Ren Nutr. 2005;15:332–44. 10.1016/j.jrn.2005.04.001 16007563

[22] Roos AN, Westendorp RG, Brand R, Souverijn JH, Frolich M, Meinders AE (1995). Predictive value of tetrapolar body impedance measurements for hydration status in critically ill patients. Intensive Care Med.

[23] Foley K, Keegan M, Campbell I, Murby B, Hancox D, Pollard B (1999). Use of single-frequency bioimpedance at 50 kHz to estimate total body water in patients with multiple organ failure and fluid overload. Crit Care Med.

[24] Jacobs DO (1996). Use of bioelectrical impedance analysis measurements in the clinical management of critical illness. Am J Clin Nutr.

[25] Kellum JA, Lameire N, Aspelin P, Barsoum RS, Burdmann EA, Goldstein SL, Herzog C a, Joannidis M, Kribben A, Levey AS, MacLeod AM, Mehta RL, Murray PT, Naicker S, Opal SM, Schaefer F, Schetz M, Uchino S. KDIGO Clinical Practice Guideline for Acute Kidney Injury. Kidney Int Suppl. 2012;2:1–138.

[26] Dellinger R, Levy M, Rhodes A (2013). Surviving Sepsis Campaign: international guidelines for management of severe sepsis and septic shock, 2012. Intensive Care.

[27] Valle R, Aspromonte N, Milani L, Peacock FW, Maisel AS, Santini M, Ronco C. Optimizing fluid management in patients with acute decompensated heart failure (ADHF): the emerging role of combined measurement of body hydration status and brain natriuretic peptide (BNP) levels. Heart Fail Rev. 2011;16:519–29. 10.1007/s10741-011-9244-4 PMC3151484 21604179

[28] Guyton AC (1991). Textbook of Medical Physiology.

[29] Chan C, McIntyre C, Smith D, Spanel P, Davies SJ (2009). Combining near-subject absolute and relative measures of longitudinal hydration in hemodialysis. Clin J Am Soc Nephrol.

[30] Jones SL, Tanaka A, Eastwood GM, Young H, Peck L, Bellomo R, Mårtensson J. Bioelectrical impedance vector analysis in critically ill patients: a prospective, clinician-blinded investigation. Crit Care. 2015;19:290. 10.1186/s13054-015-1009-3 PMC4531396 26260579

[31] Rivers E, Nguyen B, Havstad S, Ressler J, Muzzin A, Knoblich B, Peterson E, Tomlanovich M. Early goal-directed therapy in the treatment of severe sepsis and septic shock. N Engl J Med. 2001;345:1368–77. 10.1056/NEJMoa010307 11794169

